# Interleukin-10-819 promoter polymorphism in association with gastric cancer risk

**DOI:** 10.1186/1471-2407-12-102

**Published:** 2012-03-21

**Authors:** Huiping Xue, Bing Lin, Jianfu An, Yuyuan Zhu, Gang Huang

**Affiliations:** 1 Division of Gastroenterology and Hepatology, Shanghai Jiaotong University School of Medicine Renji Hospital, Shanghai Institution of Digestive Disease, Shanghai, People's Republic of China; 2 Key Laboratory of Gastroenterology & Hepatology, Ministry of Health(Shanghai Jiaotong University), Shanghai, People's Republic of China; 3 Division of Nutrition, Zhongshan Hospital, Fudan University School of Medicine, Shanghai, People's Republic of China; 4 Division of Bioinformatics, Shanghai Jiaotong University School of Medicine,, Shanghai, People's Republic of China; 5 Department of Nuclear Medicine, Shanghai Jiaotong University School of Medicine Renji Hospital, Shanghai, People's Republic of China

**Keywords:** Interleukin 10, Gene, Single nucleotide polymorphism, Association, Gastric cancer

## Abstract

**Background:**

Potential functional allele T/C single nucleotide polymorphism (SNP) of Interleukin 10 (IL-10) promoter -819 (rs1800871) has been implicated in gastric cancer risk. We aimed to explore the role of T/C SNP of IL-10 -819 in the susceptibility to gastric cancer through a systematic review and meta-analysis.

**Methods:**

Each initially included article was scored for quality appraisal. Desirable data were extracted and registered into databases. 11 studies were ultimately eligible for the meta-analysis of IL-10 -819 T/C SNP. We adopted the most probably appropriate genetic model (recessive model). Potential sources of heterogeneity were sought out via subgroup and sensitivity analyses, and publication biases were estimated.

**Results:**

IL-10 -819 TT genotype is associated with the overall reduced gastric cancer risk among Asians and even apparently observed among high quality subgroup Asians. IL-10-819 TT genotype is not statistically associated with the overall reduced gastric cancer susceptibility in persons with *H. pylori *infection compared with controls without *H. pylori *infection. IL-10 -819 TT genotype is reversely associated with diffuse-subtype risk but not in intestinal-subtype risk. IL-10 -819 TT genotype is not reversely associated with non-cardia or cardia subtype gastric cancer susceptibility.

**Conclusions:**

IL-10 -819 TT genotype seems to be more protective from gastric cancer in Asians. Whether IL-10 -819 TT genotype may be protective from gastric cancer susceptibility in persons infected with *H. pylori *or in diffuse-subtype cancer needs further exploring in the future well-designed high quality studies among different ethnicity populations. Direct sequencing should be more used in the future.

## Background

Nowadays, worldwide gastric cancer incidence has decreased but its mortality still ranks second [1-3]. In Asia [4], especially China [5], gastric cancer constitutes the top lethal malignancy. As is widely known, infectious, dietary, environmental, and genetic factors are implicated in gastric carcinogenesis, but only a minority of persons exposed to risk factors such as *Helicobacter pylori *(*H. pylori*) infection ultimately develop gastric cancer [6], which implies that host genetic susceptibility plays an important role in developing gastric cancer [7-9]. Such various susceptibilities could be explained, in part, by single nucleotide polymorphisms (SNPs) of susceptible genes [7-9]. During the long pathogenesis from chronic gastritis to gastric cancer spawned by *H. pylori *infection, host-activated neutrophils and mononuclear cells can produce not only proinflammatory cytokines such as interleukin (IL)-1β, IL-6, IL-8 and tumor necrosis factor (TNF)-α but also anti-inflammatory cytokines like IL-10. Rivetingly, the level of IL-10 besides those of IL-1 and TNF-α could also be elevated in gastric mucosa infected with *H. pylori*.

IL-10, a potent pleiotropic cytokine, has the dual ability to immunosuppress or immunostimulate anti-cancer properties [10]. Interleukin-10 inhibits the production of pro-inflammatory cytokines by inhibition of T-helper 1 (Th1) lymphocytes and stimulation of B lymphocytes and Th2 lymphocytes and thus downregulates the inflammatory response [10-12]. The human IL-10 gene, located on chromosome 1q31-32, consists of five exons and four introns and one of polymorphisms is reported in its promoter region at position -819 C/T SNP [13].

In 2003, Wu MS et al. [14] first published their study on IL-10-819 C/T SNP. Since then, researchers have consecutively reported associations of IL-10-819 C/T SNP with the susceptibility to gastric cancer, but with mixed or conflicting results [15-25]. Up to now, there has been only one published meta-analysis article focusing on IL-10-819 C/T SNP [26], but that meta-analysis failed to adopt the most likely appropriate genetic model, and thus the authentic values of statistical results could be compromised.

Accordingly, the aim of our meta-analysis was to shed more light, using the most appropriate genetic model, on the role of IL-10-819 C/T SNP in the risk of developing gastric cancer and to identify possible sources of heterogeneity among the eligible studies.

## Methods

### Search strategy

A systematic literature search was performed for articles regarding IL-10-819 C/T SNP associated with the risk of developing gastric cancer. The MEDLINE, EMBASE databases, Chinese National Knowledge Infrastructure (CNKI), Web of Science, and BIOSIS databases were used simultaneously with the combination of terms "Interleukin 10", "IL-10", "interleukin", or "cytokine"; "gene"; "polymorphism", "variant", or "SNP"; and "gastric cancer", "gastric carcinoma", "diffuse gastric cancer" or "stomach cancer" from January 2000 to September 2011. The search was performed without any restriction on language. The scope of computerized literature search was expanded according to the reference lists of retrieved articles. The relevant original articles were also sought manually.

### Study selection

Studies concerning the association of IL-10-819 C/T SNP with the risk of developing gastric cancer were included if the following conditions were met: (i) any study described the association of IL-10-819 C/T SNP with gastric cancer; (ii) any study reported the numbers of both controls and gastric cancer cases; (iii) results were expressed as odds ratio (OR) with 95% confidence intervals (CI); and (iv) studies were case-control or nested case-control ones.

### Methodological quality appraisal

To identify high-quality studies, we mainly adopted predefined criteria for Quality Appraisal initially proposed by Thakkinstian et al. [27], adapted by Camargo et al. [28], and refined by Xue et al. [7-9]. The criteria (seen in Additional file 1: Table S1) cover credibility of controls, representativeness of cases, consolidation of gastric cancer, genotyping examination, and association assessment [7-9]. Methodological quality was independently assessed by two investigators (B. Lin and J. An). Disagreements were resolved through discussion. Scores ranged from the lowest zero to the highest ten. Articles with the score lower than 6.5 were considered "low or moderate quality" ones, whereas those no lower than 6.5 were thought of as "high quality" ones.

### Data extraction

The following data from each article were extracted: authors, year of publication, country, ethnicity of participants (categorized as Caucasians, Asians, Latinos, etc.), study design, source of controls, number of controls and of cases, genotyping method, distribution of age and gender, Lauren's classification (intestinal, diffuse, or mixed), and anatomical classification (cardia or non-cardia cancer).

The data were extracted and registered into two databases independently by two investigators (B.Lin and J An) who were blind to journal names, institutions or fund grants. Any discrepancy between these two investigators was resolved by the third investigator (H. Xue), who participated in the discussion with them and made an ultimate decision.

### Statistical analysis

All statistical analyses were performed using STATA statistical software (Version 10.1, STATA Corp, College Station, TX). Two-sided Ps < 0.05 were considered statistically significant. HWE in controls was calculated again in our meta-analysis. The chi-square goodness of fit was used to test deviation from HWE (significant at the 0.05 level). Odds ratios (OR) and 95% confidence intervals (95% CI) were employed to assess the strength of associations between IL-10-819 T/C SNP with gastric cancer risk. OR_1_, OR_2_, and OR_3 _regarding IL-10-819 T/C SNP were calculated for genotypes TT versus CC, CT versus CC, and TT versus CT, respectively.

The above pairwise differences were used to determine the most appropriate genetic model. If OR_1 _= OR_3 _≠ 1 and OR_2 _= 1, then a recessive model is suggested. If OR_1 _= OR_2 _≠ 1 and OR_3 _= 1, then a dominant model is implied. If OR_2 _= 1/OR_3 _≠ 1 and OR_1 _= 1, then a complete overdominant model is suggested. If OR_1 _> OR_2 _> 1 and OR_1 _> OR_3 _> 1, or OR_1 _< OR_2 _< 1 and OR_1 _< OR_3 _< 1, then a codominant model is indicated [29]. If a dominant model was indicated, the original grouping was collapsed and the new group of T carriers (TT + CT) was compared with CC genotype; if a recessive model was suggested, TT was compared to the group of CC plus CT; if a complete overdominant model was implied, the group of TT plus CC was compared with CT; or if a codominant model was insinuated, TT was compared with CT and with CC, respectively.

The Q statistic was used to test for heterogeneity among the studies included in the meta-analysis. A fixed-effects model, using Mantel-Haenszel (M-H) method, was used to calculate the pooled ORs when homogeneity existed on the basis of Q-test p value no less than 0.1. By contrast, a random-effects model, using DerSimonian and Laird method (D + L), was utilized if there was heterogeneity based on Q-test p value less than 0.1. The significance of pooled ORs was tested by Z test (P < 0.05 was considered significant).

Sensitivity analysis was performed, in which the meta-analysis estimates were computed after every one study being omitted in each turn.

Finally, publication bias was assessed by performing funnel plots qualitatively, and estimated by Begg's and Egger's tests quantitatively.

## Results

### Literature search and study selection

After comprehensive searching, a total of 242 articles (236 in English and 6 in Chinese) were retrieved. 230 articles were initially excluded after being read by their respective title and abstract (203 not related gene polymorphism, 6 the same gene but not related locus polymorphisms, 7 related to other gastrointestinal diseases other than gastric cancer, 2 related to precancerous gastric lesions, 1 meta-analysis paper, 1 colorectal carcinoma, 7 related to the effects of bacterial or viral factors (2 Epstein-Barr virus, 5 Helicobacter pylori), 3 unrelated reviews). In our meta-analysis were initially included altogether 12 studies [14-25] which catered to the inclusion criteria, and these 12 full-text articles then were considered for further evaluation. 1 article was further excluded due to its lacking normal healthy controls [25]. Those 11 studies seemed appropriate to the meta-analysis of the associations with gastric cancer regarding IL-10-819 T/C SNP. Two studies [20,24] were deviated from HWE through our calculation. Generally speaking, any study that deviated from Hardy-Weinberg equilibrium should have been removed; however, considering that the numbers of participants in those two studies were large and given that sensitivity analyses would be conducted, we remained those two studies in our meta-analysis.

Thus, 11 studies [14-24] with a total of 4008 controls and 1490 cases were ultimately eligible for the meta-analysis of IL-10-819 T/C SNP. The corresponding characteristics were seen in Table 1. The flow chart of literature search and study selection was seen in Figure 1.

**Table 1:** Study Characteristics of genotypes in gastric cancer cases and controls in the analysis of Interleukin-10-819 Promoter Genetic Polymorphisms

First author	Year of publication	Quality assessment scores	Genotyping method	Total sample size	Number of controls	Number of cases	Study location	Ethnic group	P values for HWE	Controls, genotypes(n)			All Cases, genotypes(n)		
										CC	CT	TT	CC	CT	TT
Wu **MS**	2003	7	Direct sequencing	450	230	220	China	Asians	0.231397685	20	83	127	27	105	88
Savage SA^#^	2004	5	ABI Genetic Analyzer	466	382	84	China	Asians	0.314869012	49	163	170	9	38	37
Alpízar-Alpízar W	2005	6	Pyrosequencing	90	45	45	Costa Rica	Latinos	0.08326454	18	24	3	25	16	4
Zambon CF^^^	2005	5	TaqMan	773	644	129	Italy	Caucasians	0.696436614	353	245	46	70	42	17
Kamangar F^#^¶^	2006	8	TaqMan	250	152	98	Finland	Caucasians	0.66272429	80	62	10	58	35	5
Sugimoto M^#^*¶**+**^	2007	6.5	ASP	273	168	105	Japan	Asians	0.194224595	9	73	86	6	57	42
Crusius JB^#^^	2008	8.5	ABI real-time PCR	1323	1094	229	European	Caucasians	0.02386503	636	378	80	145	72	12
Xiao H	2009	6	RFLP	844	624	220	China	Asians	0.718880427	69	283	272	20	100	100
Ko KP	2009	7	SNaPshot	409	326	83	Korea	Asians	0.038333741	37	121	168	11	33	39
Su SP	2010	6.5	RFLP	143	100	43	China	Asians	0.433216715	6	43	51	4	21	18
Liu J**^+^**	2011	6.5	RFLP	477	243	234	China	Asians	0.772829993	28	106	109	39	96	99

#Data of cardia-subtype gastric cancer were accessible; ^ Data of noncardia-subtype gastric cancer were accessible; * Data of sporadic diffuse-subtype gastric cancer were accessible; ¶ Data of intestinal-subtype gastric cancer were accessible. +Data of the status of *Helicobacter pylori *of gastric cancer were accessible. RFLP: Restriction fragment length polymorphisms; TaqMan: 5'nuclease polymerase chain reaction assays; Pyrosequencing: a method of DNA sequencing (determining the order of nucleotides in DNA) based on the "sequencing by synthesis" principle. It differs from Sanger sequencing, in that it relies on the detection of pyrophosphate release on nucleotide incorporation, rather than chain termination with dideoxynucleotides; Direct sequencing: method of methylation analysis using bisulfite-treated DNA utilized PCR and standard dideoxynucleotide DNA sequencing to directly determine the nucleotides resistant to bisulfite conversion; ASP: the allele specific primer-polymerase chain reaction (ASP-PCR) method; SNaPshot: the SNaPshot assay which provides detection of certain SNPs

**Figure 1:**
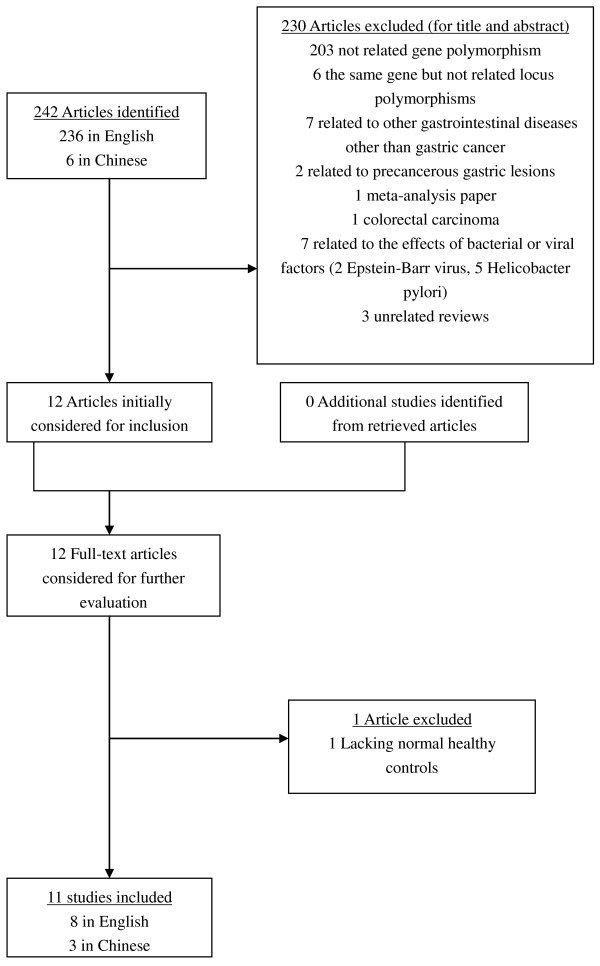
**The flow chart of literature search and study selection**.

### Overall meta-analysis among different ethnicity populations

**Figure 2:**
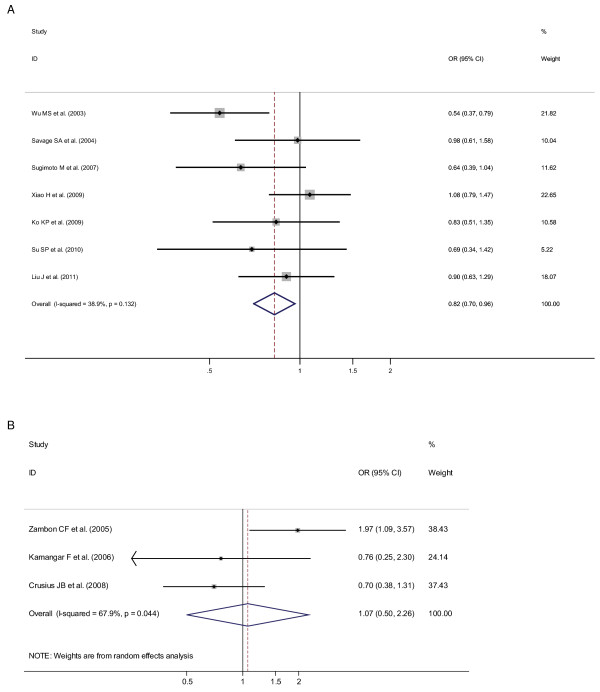
**Odds ratios (ORs) for associations between IL-10-819 T/C SNP and gastric cancer risk (TT vs CT-plus-CC) among different ethnicity populations, in order of increasing publication year, 2003-2011**. Studies were entered into the meta-analysis sequentially by year of publication. The sizes of the squares indicate the relative weight of each study. Bars, 95% confidence interval (CI). A) among Asians; B) among Caucasians.

OR_1 _(p value), OR_2 _(p value), and OR_3 _(p value) of IL-10-819 T/C SNP for overall ethnicities were 0.87 (p = 0.202), 0.86 (p = 0.083), and 0.88 (p = 0.095), respectively, hardly insinuating a probably suitable genetic model effect of putative protective T allele. Meanwhile, after ethnicity subgroup analysis, OR_1 _(p value), OR_2 _(p value), and OR_3 _(p value) of IL-10-819 T/C SNP among Asians were 0.81 (p = 0.120), 0.95 (p = 0.734), and 0.83 (p = 0.027), respectively, suggesting a recessive genetic model effect of putative protective T allele (OR_1 _= OR_3 _< 1 and OR_2 _= 1). Thus, the genotype TT was compared with the combined genotype CT-plus-CC. As in Figure 2, for overall gastric cancer a statistically significant finding could be noted among Asians (Figure 2A) but not among Caucasians (Figure 2B) from the facts that the pooled ORs (95% CI, p value) were 0.82 (0.70-0.96, p = 0.015) for the former and 1.07 (0.50-2.26, p = 0.869) for the latter. Only one included study [16] dealt with latino population or mixed ethnicity, so pooled ORs could not be calculated among latino population in our meta-analysis.

### Further subgroup analysis

**Figure 3:**
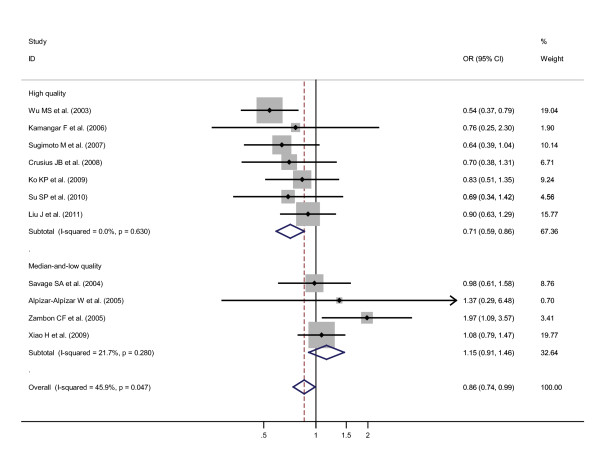
**Odds ratios (ORs) for associations between IL-10-819 T/C SNP and gastric cancer risk (TT vs CT-plus-CC) among high quality subgroup participants regardless of ethnicities and among median-and-low quality subgroup participants regardless of ethnicities**. The sizes of the squares indicate the relative weight of each study. Bars, 95% confidence interval (CI).

Specific data for IL-10-819 T/C SNP were classified in accordance with the quality appraisal scores, into high quality (scores no less than 6.5) and median-and-low quality (scores less than 6.5) subgroups among different ethnicities. A statistically significant reverse association was only witnessed in Asians high quality subgroup but not in Asians median-and-low quality subgroup, on the grounds that the pooled ORs (95% CIs, p value) were 0.71 (0.58-0.87, p = 0.001) for the former and 1.05 (0.81-1.36, p = 0.719) for the latter. The pooled ORs (95% CIs, p value) among Caucasians median-and-low quality subgroup could not be calculated due to only 1 included study. The pooled ORs (95% CIs, p value) among Caucasians high quality subgroup were 0.71 (0.42-1.23, p = 0.225). If Asians high quality subgroup and Caucasians high quality subgroup were combined, and Asians median-and-low quality subgroup, Caucasians median-and-low quality subgroup, and Latinos median-and-low quality subgroup were also combined, the pooled ORs (95% CIs, p value) were 0.71 (0.59-0.86, p = 0.000) for the former (the combined high quality subgroup) and 1.15 (0.91-1.46, p = 0.240) for the latter (the combined median-and-low quality subgroup), based on the recessive genetic model in our initial option (Figure 3). To further confirm the recessive genetic model, the above OR_1 _(p value), OR_2 _(p value), and OR_3 _(p value) of IL-10-819 T/C SNP in the combined high quality subgroup for overall ethnicities were 0.64 (p = 0.002), 0.82 (p = 0.065), and 0.75 (p = 0.004), respectively, again indicating a recessive genetic model effect of putative protective T allele (OR_1 _= OR_3 _< 1 and OR_2 _= 1).

When gastric cancer was classified into non-cardia (or distal) and cardia subtypes, no statistically significant findings were found among non-cardia subtype or among cardia subtype on the grounds that the pooled ORs (95% CIs, p value) were 0.82 (0.38-1.76, p = 0.603) among non-cardia subtype and 1.02 (0.67-1.56, p = 0.913) among cardia subtype. In terms of pathology, gastric cancer could be classified into intestinal, diffuse, or mixed subtypes, and no statistically significant finding was observed in intestinal-subtype cancer but in diffuse-subtype cancer, for the pooled ORs (95% CIs, p value) were 0.78 (0.48-1.27, p = 0.318) in the former and 0.32 (0.12-0.84, p = 0.021) in the latter.

In terms of *H. pylori *infection status, no statistically significant reverse association was noted among either *H. pylori *positive cancer patients compared with *H. pylori *negative controls or among *H. pylori *positive cancer patients compared with *H. pylori *positive controls, for pooled ORs (95% CIs, p value) were 0.64 (0.39-1.04, p = 0.072) in the former and 0.90 (0.63-1.29, p = 0.575) in the latter, but the p value was approximate to 0.05 in the former.

And when genotyping techniques were considered, a statistically significant finding was noted in direct sequencing subgroup but not in any other genotyping technique subgroup. In the direct sequencing, TaqMan, ABI Genetic Analyzer, Pyrosequencing, Snapshot, RFLP, ASP, and ABI real-time PCR genotyping technique subgroups, pooled ORs (95% CIs, p value) were 0.54 (0.37-0.79, p = 0.001), 1.54 (0.91-2.60, p = 0.106), 0.98 (0.61-1.58, p = 0.939), 1.37 (0.29-6.48, p = 0.695), 0.83 (0.51-1.35, p = 0.460), 0.96 (0.77-1.21, p = 0.754), 0.64 (0.39-1.04, p = 0.072), and 0.70 (0.38-1.31, p = 0.265), respectively.

### Sensitivity analysis

Meta-analyses were conducted repeatedly when each particular study had been removed. The results indicated that fixed-effects estimates and/or random-effects estimates before and after the deletion of each study were similar at large, suggesting moderate to high stability of the meta-analysis results. The most influencing single study on the overall pooled estimates seemed to be the study conducted by Wu et al. [14], the sensitivity analysis, however, indicated moderate stability of the results from the facts that the ORs (95% CI, p value) for all ethnicity were 0.86 (0.74-0.99, p = 0.037) before the removal of that study and 0.94 (0.80-1.12, p = 0.488) after the removal of that study. In view of the study [20] conducted by Crusius JB et al. which is deviated from HWE, the ORs (95% CI, p value) were 0.86 (0.74-0.99, p = 0.037) before the removal of that study and 0.87 (0.75-1.01, p = 0.063) after the removal of that study for the all ethnicity, indicating moderate to high stability of the results. Similarly, after the removal of the study [24] conducted by Ko KP et al., also deviated from HWE, the OR (95% CI, p value) became 0.86 (0.74-1.00, p = 0.050), indicating high stability of the results (The illustrating figures were omitted due to the length of paper).

### Cumulative meta-analysis

**Figure 4:**
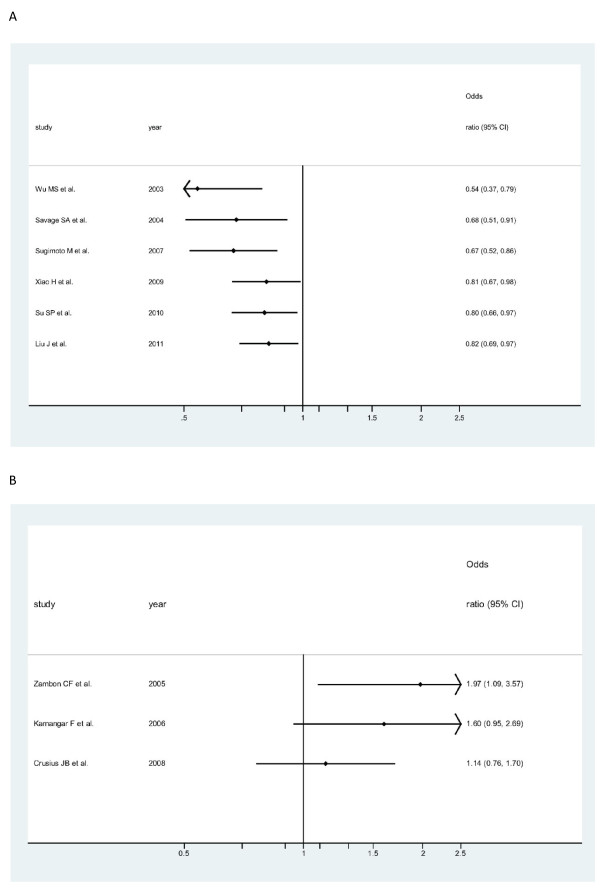
**Cumulative meta-analysis of associations between the IL-10-819 TT genotype, as compared with the combined CT-plus -CC genotype, and gastric cancer risk among different ethnicity populations sorted by publication year and the total sample size**. Horizontal line, the accumulation of estimates as each study was added rather than the estimate of a single study. A) among Asians; B) among Caucasians.

Cumulative meta-analyses of IL-10-819 T/C SNP association were also conducted among Asians (Figure 4A) and among Caucasians (Figure 4B) via the assortment of total number of sample size. As shown in Figure 4A, the inclination toward significant reverse associations with overall gastric cancer, though somewhat undulated, was obviously seen among Asians, whereas in Figure 4B, the seeming opposite tendency was observed among Caucasians.

### Publication bias analysis

**Figure 5:**
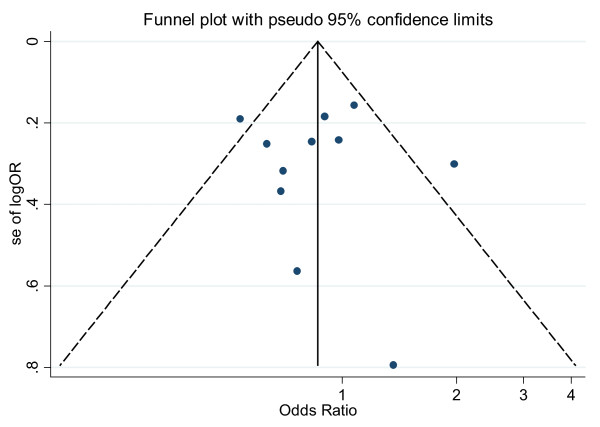
**Funnel plot of publication bias for IL-10-819 SNP (TT vs CT-plus-CC)**. Note: Funnel plot with pseudo 95% confidence limits was used.

Publication bias was preliminarily examined by funnel plots qualitatively and estimated by Begg's and Egger's tests quantitatively. Its funnel plot (Figure 5) showed that dots nearly symmetrically distributed, predominantly within pseudo 95% confidence limits. P values were 1.000 in Begg's test and 0.897 in Egger's test, separately, also suggesting no publication bias.

## Discussion

In our meta-analysis, a statistically significant finding could be noted with the overall reduced risk of gastric cancer among Asians but not among Caucasians (TT vs CT-plus-CC); the opposite tendency toward the risk of gastric cancer could also be observed between Caucasians and Asians via cumulative meta-analysis sorted by publication time and total sample size. Thus, IL-10-819 TT genotype may seem to be more protective from overall gastric cancer susceptibility among Asians. To be sure, the different or even conflicting risk associations, if so, among different ethnicities should be further meticulously investigated and confirmed in the future.

Our further subgroup analyses also indicate that a statistically significant reverse association was witnessed in Asians high quality subgroup but not in Asians median-and-low quality subgroup; the reverse association tendency was also observed in Caucasians high quality subgroup, although the statistical significance could not be reached. The consistent reverse association trend between Asians high quality subgroup and Caucasians high quality subgroup could be apparently seen. The strong statistical significant reverse association could be found among the combined high quality subgroup regardless of ethnicities based on the recessive genetic model in our meta-analysis. Furthermore, the recessive genetic model was confirmed through the recalculation of OR_1 _(p value), OR_2 _(p value), and OR_3 _(p value) in the combined high quality subgroup regardless of ethnicities. Therefore, it should be advocated that more rigorous high-quality studies should be designed in the future so as to accurately explore the real associations between IL-10-819 TT genotype and gastric cancer susceptibility among different ethnicities.

Additionally, 4[17-20] out of 11 eligible studies were dealt with noncardia-subtype gastric cancer and 4 [15,18-20] with cardia-subtype gastric cancer. No statistically significant findings could be noted with either subtype (TT vs CT-plus-CC). 2 studies [18,19] in our meta-analysis were dealt with pathologically intestinal-subtype gastric cancer and only 1 [19] out of 11 studies was dealt with pathologically diffuse-subtype gastric cancer. No statistically significant finding could be noted in intestinal-subtype but in diffuse- subtype cancer (TT vs CT-plus-CC). As is known, cardia-subtype gastric cancer differs from noncardia-subtype gastric cancer in etiology, pathology, carcinogenesis, and/or prognosis [30-32], so is intestinal-subtype cancer versus diffuse-subtype cancer. It could be said that the indiscriminate combination of cardia-subtype and noncardia-subtype cases or intestinal-subtype and diffuse-subtype cases in the majority of eligible studies may mask or at least underestimate the strength of the real associations [7-9].

Furthermore, it was reported that gastric cancer develops in those with *H. pylori *infection rather than in uninfected ones [33]. In our meta-analysis, no statistically significant reverse association with gastric cancer was found either among *H. pylori *positive cancer patients compared with *H. pylori *negative controls or among *H. pylori *positive cancer patients compared with *H. pylori *positive controls (TT vs CT-plus-CC), but the p value in the former was approximate to 0.05, insinuating that IL-10-819 TT genotype may seem to be more protective from overall gastric cancer susceptibility in persons infected with *H. pylori*. Certainly, the real association between *H pylori *infection and IL-10-819 TT genotype and gastric cancer susceptibility should be further meticulously investigated in the future.

With the coming of new genotyping technologies like seminested polymerase chain reaction, TaqMan allelic discrimination test, direct sequencing, the allele specific primer-polymerase chain reaction, pyrosequencing, or real-time PCR, we may witness an upsurge of genetic association studies in the future. In our meta-analysis, a statistically significant reverse association with gastric cancer susceptibility was only noted in direct sequencing technique subgroup but not in any other genotyping subgroup. The fact that the most significant result can be witnessed in direct sequencing technology in our meta-analysis is not necessarily a valid reason to demonstrate that other technologies cannot be used. Certainly, for a novel genotyping technique to be employed for the study of a particular genetic polymorphism, this technology should better be confirmed using direct sequencing. In that case, this new technology can be seen as valid as direct sequencing. Or the sensitivity and specificity of those genotyping techniques need to be explored so as to seek out optimal approaches which could minimize the genotyping errors [7-9]. Our opinion is that direct sequencing should be more used in future well-designed studies among different ethnicity populations.

And the mechanism of the influence of IL-10-819 SNP on carcinogenesis is still unknown, but it has been reported that IL-10 SNPs may influence immune function through modulating the activities of the NK cell, T cells, and macrophages and thus alter the disease progression [34]. Additionally, another investigated SNP at position -1082 (A/G) was reported to be significantly increased in prostate cancer patients and its action, together with VEGF and IL-8, was suspected to possibly influence cancer angiogenesis [35]. Whether IL-10-819 SNP may also influence cancer angiogenesis is worthy of further investigating in the future.

Finally, the strength of our meta-analysis could be summarized as follows. We sought to find as many publications as we could by means of various searching approaches. Any study that appeared to deviate from HWE was not excluded mechanically in our meta-analysis unless there are other convincing grounds for doubting the quality of the study [36]. We laid more emphasis on assessing biases across studies and pinpointing the potential sources of heterogeneity via subgroup and sensitivity analyses. More importantly, we have made great efforts to stratify ethnicity into Asians subgroup and Caucasian subgroup in accordance with accessible data. In particular, we have conducted overall meta-analysis among different ethnicity populations to carefully choose the most likely appropriate genetic model. We also have stratified the included studies through other subgroup analyses like anatomic classification, pathologically Lauren's classification, *H. pylori *infection status, sample size, and quality appraisal scores. We comprehensively assessed the publication biases using several means like Begg's and Egger's tests as well as funnel plot tests. In view of this, we convince that the results of our meta-analysis, in essence, are sound and reliable.

Certainly, there are some unavoidable limitations in our meta-analysis. Firstly, the information about overall gastric cancer susceptibility is predominantly provided, while other information about pathologic subtypes or anatomic subtypes of gastric cancer is less provided. Thus, the specific subtype results should be considered with caution. Secondly, with the merely published studies included in our meta-analysis, publication bias is very likely to occur, though no statistically significant publication bias is found in our meta-analysis. Thirdly, slight to moderate heterogeneity could be witnessed among the included studies. So as to minimize the potential bias, we designed a rigorous protocol before conducting our meta-analysis, and performed a scrupulous search for published studies using explicit methods for study selection, data extraction, statistical analysis, adoption of the most appropriate genetic model with extreme caution and sensitivity analysis.

## Conclusions

In conclusion, IL-10-819 TT genotype may seem to be more protective from overall gastric cancer susceptibility among Asians and even more protective in high quality subgroup Asians. IL-10-819 TT genotype is not statistically associated with gastric cancer susceptibility in persons infected with *H. pylori*. IL-10-819 TT genotype is not associated with pathologic intestinal subtype but in diffuse subtype and not with anatomic subtypes (non-cardia or cardia) of gastric cancer susceptibility in our meta-analysis. Direct sequencing should be more used in future well-designed high quality studies among different ethnicities or populations.

## Competing interests

The authors declare that they have no competing interests.

## Authors' contributions

Data were acquired by LB and AJ; Data were analyzed by XH, LB, AJ, ZY, and HG; This Manuscript was written by XH and revised by HG; and Statistical analysis was made by XH, AJ, LB and ZY. HG and XH designed, coordinated and supervised this whole work. All authors read and approved the final manuscript.

## Pre-publication history

The pre-publication history for this paper can be accessed here:

http://www.biomedcentral.com/1471-2407/12/102/prepub

## Supplementary Material

Additional file 1 **Table S1**. Scales for Quality Assessment. Click here for file
